# Meningeal Melanomatosis: A Challenge for Timely Diagnosis

**DOI:** 10.1155/2015/948497

**Published:** 2015-01-14

**Authors:** Giulia Berzero, Luca Diamanti, Anna Luisa Di Stefano, Paola Bini, Diego Franciotta, Ilaria Imarisio, Paolo Pedrazzoli, Lorenzo Magrassi, Patrizia Morbini, Lisa Maria Farina, Stefano Bastianello, Mauro Ceroni, Enrico Marchioni

**Affiliations:** ^1^C. Mondino National Neurological Institute, Via Mondino 2, 27100 Pavia, Italy; ^2^Neuroscience Consortium, University of Pavia, Monza Policlinico and Pavia Mondino, 27100 Pavia, Italy; ^3^AP-HP, Groupe Hospitalier Pitié-Salpêtrière, Service de Neurologie Mazarin, 75013 Paris, France; ^4^Oncology Unit, Policlinico San Matteo Foundation IRCCS, 27100 Pavia, Italy; ^5^Neurosurgery Unit, Policlinico San Matteo Foundation IRCCS, 27100 Pavia, Italy; ^6^Department of Molecular Medicine, Unit of Pathology, University of Pavia and Policlinico San Matteo Foundation IRCCS, 27100 Pavia, Italy; ^7^Department of Brain and Behavioral Sciences, University of Pavia, 27100 Pavia, Italy

## Abstract

Neoplastic meningitis is a central nervous system complication that occurs in 3–5% of patients with cancer. Although most commonly seen in patients with disseminated disease, in a small percentage of patients, it may be the initial manifestation of cancer or even primitive in origin. In the absence of cancer history, the diagnosis of neoplastic meningitis may be challenging even for expert neurologists. Prognosis is poor, with a median overall survival of weeks from diagnosis. In the retrospective study herein, we described three cases of meningeal melanomatosis in patients without previous cancer history. The patients were diagnosed with significant delay (17 to 47 weeks from symptom onset) mainly due to the deferral in performing the appropriate testing. Even when the diagnosis was suspected, investigations by MRI, cerebrospinal fluid, or both proved at times unhelpful for confirmation. Prognosis was dismal, with a median survival of 4 weeks after diagnosis. Our observations are a cue to analyze the main pitfalls in the diagnosis of neoplastic meningitis in patients without cancer history and emphasize key elements that may facilitate early diagnosis.

## 1. Introduction

Neoplastic meningitis is a central nervous system complication that occurs in 3–5% of patients with cancer [1], and it is most commonly seen in patients with disseminated progressive systemic disease due to spread of malignant cells to the leptomeninges. The most common primary tumors to metastasize to the meninges are lung cancer (9–25% of patients) [2] and melanoma (23%) [3], due to a distinctive neurotropism. More rarely neoplastic meningitis is the initial manifestation of systemic cancer (5–10%) [1] or it is primitive in origin, as it occurs in primary leptomeningeal melanomatosis [4]. In patients without cancer history, diagnosis may be challenging even for expert neurologists due to the lack of specific signs and symptoms. Prognosis of neoplastic meningitis is poor, as most untreated patients die within 1–9 weeks from diagnosis (median 3 weeks) [1, 5], as a result of neurological disease or tumor progression. The timeliness of diagnosis is crucial to start the appropriate treatment before sudden neurological deterioration.

Here, we present a retrospective series of three patients with meningeal melanomatosis without history of cancer, characterized by a dramatic diagnostic delay. We also propose an algorithm focused on the diagnosis of neoplastic meningitis in naïve patients.

## 2. Materials and Methods

We describe a retrospective series of three patients with meningeal melanomatosis recruited from our two institutions (C. Mondino National Neurological Institute and Policlinico San Matteo Foundation IRCCS, Pavia, Italy) in four years. We conducted an Internal Review Board approved study using an institutional oncological database of all patients receiving a diagnosis of meningeal melanomatosis from January 2010 to January 2014. The medical records were reviewed and clinical, biological, and radiological data collected for details.

## 3. Results

**Figure 1:**
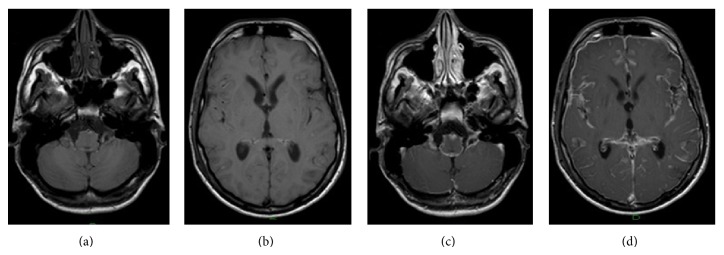
Brain diffuse leptomeningeal melanomatosis in patient 1. (a), (b): T1 weighted axial image (TR/TE 1157 ms/45 ms) shows diffuse sulcal signal hyperintensity due to melanin products which cause T1 shortening signal. (c), (d): contrast-enhanced T1-weighted volume image (TR/TE 25 ms/4,6 ms) shows prominent and extensive leptomeningeal enhancement.

**Figure 2:**
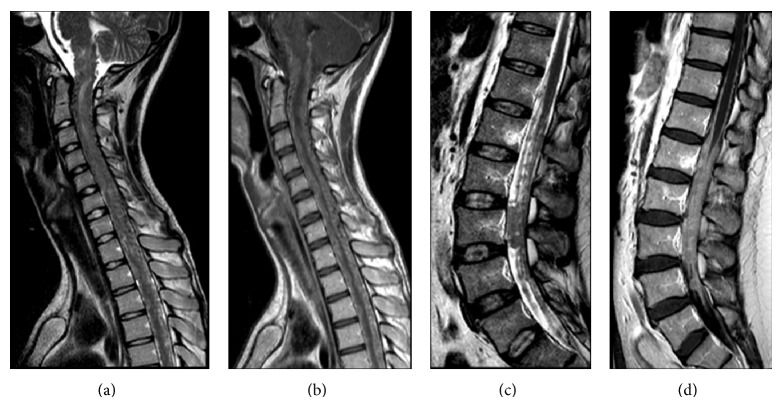
Primary leptomeningeal melanomatosis in patients 1 ((a), (b)) and 2 ((c), (d)). (a), (c): Sagittal T2-weighted images (TR/TE 3500 ms/120 ms) of cervical-dorsal spine and cauda equina show hypertrophic leptomeninges with crowded subarachnoid space and multinodular appearance of the cauda equina. (b), (d): Sagittal T1-weighted images (TR/TE 65 ms/9 ms) of cervical-dorsal spine and cauda equina show diffuse leptomeningeal enhancement and thickening.

**Figure 3:**
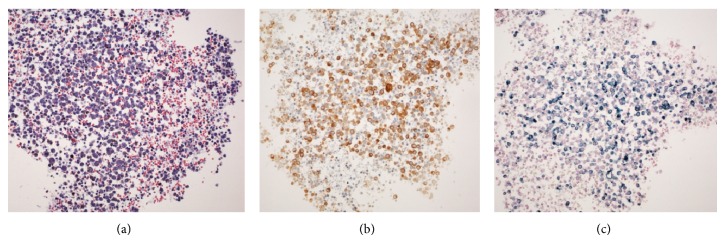
Light microscopy pictures of the cytological specimen of cerebrospinal fluid obtained from patient 1: (a) hematoxylin and eosin staining of the hypercellular sample, with large, hyperchromatic cells associated with erythrocytes; (b) atypical cells stained with Melan-A, a melanoma-specific marker; (c) Schmorl staining confirmed the presence of melanin (blue granular stain) in the cytoplasm; magnification, 20x.

**Figure 4:**
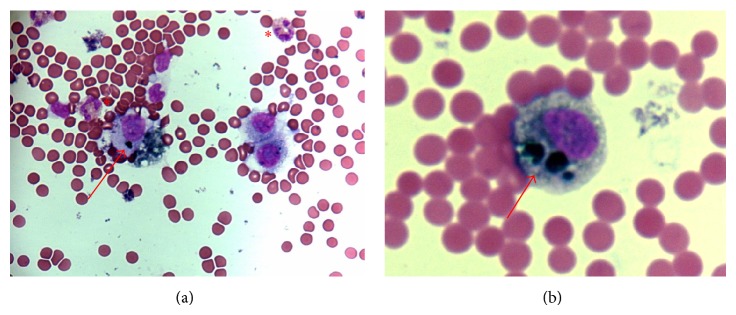
Light microscopy pictures of the cytospin of the cerebrospinal fluid cells from patient 1: (a) hematoxylin and eosin staining of large, hyperchromatic cells, along with erythrocytes, lymphoma monocytoid cells, and eosinophils (asterisks); (b) an atypical cell at larger magnification; arrows indicate granules of melanin.

The clinical and paraclinical characteristics of our three patients are summarized in Table 1. Patients were aged between 17 and 65. All patients had no previous cancer history and arrived to our centers after several neurologic evaluations. Clinical presentation included diffuse and/or multifocal neurological signs and symptoms: headache, nausea and/or vomiting, monoparesis, and cranial nerve palsies. One patient (patient 3) presented recurrent isolated confusional episodes but was completely asymptomatic in between. Electroencephalogram showed bilateral/diffuse slow abnormalities without epileptic activity in all cases. Brain MRI performed within the first 4 weeks from symptom onset was normal in both patients in whom it was acquired (pt 1 and 3). Alternatively, focal or diffuse nodular enhancement of leptomeninges and cranial nerves was documented (Figure 1). Spine MRI revealed nodular contrast enhancement of meninges, conus, and cauda, suggesting neoplastic infiltration (Figure 2). Cerebrospinal fluid (CSF) analysis showed severe blood-CSF barrier (B-CSF B) damage in all patients but inconstant pleocytosis. In patient 2, despite repeated lumbar punctures, CSF cytology remained negative and diagnosis was confirmed on leptomeningeal tissue obtained from biopsy. In all other patients, the presence of melanoma cells in the CSF (Figures 3 and 4) was eventually documented by means of repeated lumbar punctures. After the diagnosis of meningeal melanomatosis was confirmed, all patients underwent a chest-abdomen CT scan and a dermatological and an ophthalmological assessment. In two patients, the final diagnosis was of probable primary leptomeningeal melanomatosis (pt 1 and 2), while in patient 3 a cutaneous melanoma of right eyelid was documented. The diagnostic delay was remarkable in our series, with a median delay of 32 weeks from symptom onset (range: 17–47 weeks). Prognosis was dismal, with a median survival of 4.14 weeks from diagnosis (range: 2–6.29 weeks).

## 4. Discussion

**Figure 5:**
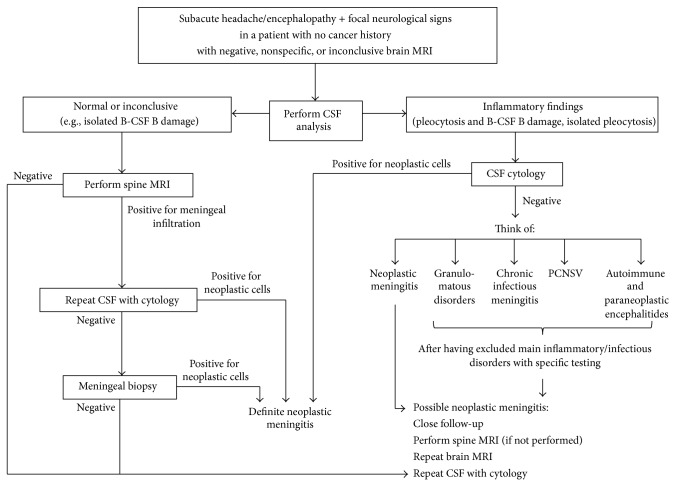
The proposed algorithm for the diagnosis of neoplastic meningitis in naïve patients. B-CSF B = blood-cerebrospinal fluid barrier, CSF = cerebrospinal fluid, and PCNSV = primary CNS vasculitis.

Although restricted, our series offers several insights into the diagnosis of neoplastic meningitis in naïve patients. These patients, without a previous cancer history, can present with diffuse/multifocal clinical signs and symptoms and represent a real diagnostic challenge. On these grounds, we propose an algorithm (Figure 5) to guide the clinician in the complex process of differential diagnosis, regarding as opening scenario a naïf patient presenting with subacute headache and/or encephalopathy plus one or more focal signs, and negative or inconclusive MRI, as we have observed in our series. In this setting, CSF analysis should be promptly performed to exclude other dysimmune/infectious disorders such as autoimmune and paraneoplastic encephalitides, primary CNS vasculitis, and chronic infectious meningitis, which can course without MRI alterations. Besides, it is important to consider that a delay in the diagnosis of the above-mentioned conditions may strongly affect final outcome and long-term sequelae.

Furthermore, even when the diagnosis of neoplastic meningitis has been suspected, paraclinical findings could be inconclusive. According to the literature, brain MRI has an estimated sensitivity of 40–60% in demonstrating meningeal neoplastic infiltration [1, 6, 7], but data correlating sensitivity of MRI to the timing of its execution are currently unavailable. In our series, brain MRI performed within the first month from onset was normal despite the clinical pattern was dominated by cerebral involvement. In the absence of meningeal contrast enhancement, dilation of the ventricular system or reduction of subarachnoid sulci may be indirect signs of neoplastic meningitis and should be valued in all cases.

CSF analysis, which was performed with remarkable delay in our series, showed a significant protein increase due to severe B-CSF B damage in all patients. Noteworthy, despite the delay and repeated sampling, in patient 2 CSF results were inconclusive for the detection of neoplastic cells, leading to performing a meningeal biopsy. Interestingly, CSF cytology can be persistently negative even in the presence of disseminated cranial and spinal disease on MRI. These data are consistent with current evidence that malignant cells are detected in the CSF in 50–70% of patients with neoplastic meningitis by initial lumbar puncture [1, 8, 9], a rate that increases with repeated sampling. In the case of normal or inconclusive CSF findings, a spine MRI may be helpful to demonstrate meningeal infiltration of cauda roots, even in the absence of spinal symptoms.

Overall, diagnostic difficulties resulted in a dramatic diagnostic delay, ranging from 17 to 47 weeks after clinical onset. These data are remarkable considering the poor short-term prognosis of these patients [10], who could access only palliative or even no treatment.

In conclusion, the difficulty in both posing the clinical suspicion and confirming the diagnosis of neoplastic meningitis contributed to the sharp diagnostic delay observed in our series. Early recognition is fundamental to make differential diagnosis and start appropriate therapies. Thus, improving the handle of these patients and the current diagnostic algorithms for neoplastic meningitis is of capital importance to offer them appropriate treatments.

## Figures and Tables

**Table 1:** Clinical and paraclinical findings in our three patients.

Pt	1			2			3	
Age/gender	17/M			55/M			65/M	

Clinical presentation at onset	Headache, nausea and vomiting, diplopia, and weight loss			Left leg monoparesis, headache, nausea and vomiting			Recurrent confusional episodes	

MRI								
Time from symptom onset (weeks)	4	30	47	8.2		12.9	1	21.4
Hydrocephalus	No	Modest dilation of ventricular system	*Tetra*-ventricular	Triventricular		Triventricular	No	Triventricular
Leptomeningeal contrast enhancement	No	Contrast not administered	Supra- and infratentorial, cranial nerves, spinal cord, conus, and cauda equina	Supratentorial, conus, and cauda equina		Supratentorial, spinal cord, conus, and cauda equina	No	Supra- and infratentorial, cranial nerves, conus, and cauda equina
Dural melanin deposits	No	No	Yes	No		No	No	Yes

CSF								
Time from symptom onset (weeks)	40.6	42.9	44.9	8.1	10.6	14.9	21.4	
Glucose (mg/dL)	—	92	83	—	—	29	8	
Proteins (mg/dL)	—	2366	2773	370	—	431	535	
Cell count (cells/uL)	4	110	174	2	<2	10	36	
Cytology	n.p.	Nonspecific	Melanoma cells	n.p.	No identifiable cells	Nonspecific	Melanoma cells	

EEG	Poorly organized background activity with bilateral slow abnormalities			Diffuse bilateral slowing			Bilateral fronto-centrotemporal slow abnormalities with left prevalence	

Extra CNS visceral met	No			No			No	

Final diagnosis	**Primary leptomeningeal melanomatosis**			**Primary leptomeningeal melanomatosis**			**Leptomeningeal carcinomatosis and cutaneous melanoma**	

Time to diagnosis (weeks)	47			17			23.4	

Clinical evolution	Confusion, visual hallucinations, partial seizures, and behavioral alterations			Urinary retention, progressive paraparesis, and visual hallucinations			Vigilance impairment, generalized seizures, headache, and backache	

Treatment	Temozolomide (I cycle)			Dacarbazine (I cycle)			None	

Overall survival (weeks)	49.1			23.4			30	

Legend to Table 1: CNS = central nervous system, CSF = cerebrospinal fluid, EEG = electroencephalogram, met = metastases, MRI = magnetic resonance imaging, n.p. = not performed, and — = not available.
